# Supplementary material for: FlexPro MD®, a Combination of Krill Oil, Astaxanthin and Hyaluronic Acid, Reduces Pain Behavior and Inhibits Inflammatory Response in Monosodium Iodoacetate-Induced Osteoarthritis in Rats
Source: Nutrients. 2020 Mar 30;12(4):956. doi: 10.3390/nu12040956 (PMC7230382; doi:10.3390/nu12040956)
Supplement: Supplementary file 1 [file nutrients-12-00956-s001.zip › nutrients-735123-supplementary.docx]

**Supplementary Figures**


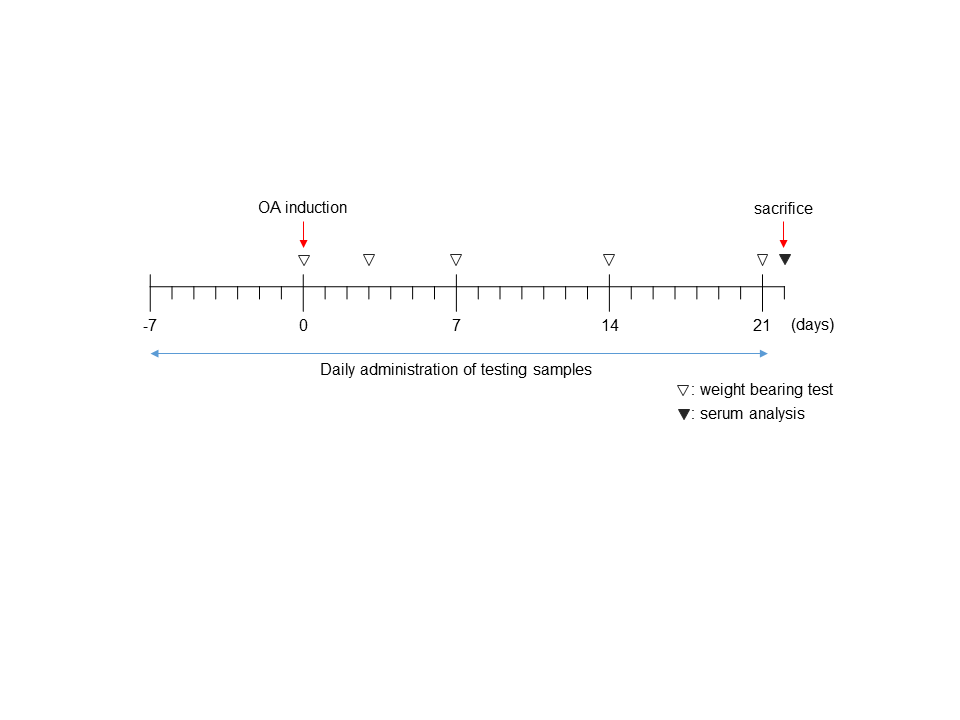


**Supplementary Figure S1. Schematic diagram of experimental design and schedule for animal experiments.** Animal experimental protocol indicating the time course for various interventions utilized during the experiments.

**
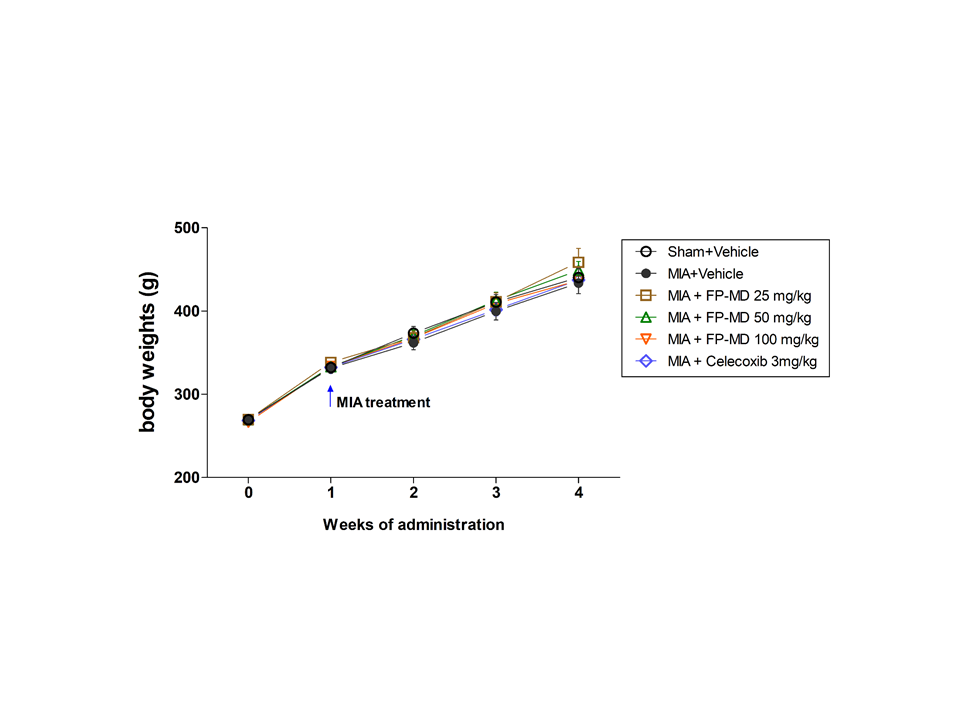
**

**Supplementary Figure 2. Effects of oral administration of FP-MD on changes in body weight gain in MIA-induced OA rats.** Body weight was continuously measured from initial to final administration. Data are expressed as the mean ± S.E.M (n=8).
